# The Tribological Behaviors in Zr-Based Bulk Metallic Glass with High Heterogeneous Microstructure

**DOI:** 10.3390/ma15217772

**Published:** 2022-11-04

**Authors:** Yubai Ma, Mei Li, Fangqiu Zu

**Affiliations:** 1 College of Chemistry and Chemical Engineering, Chongqing University, Chongqing 401133, China; 2 Liquid/Solid Metal Processing Institute, School of Materials Science and Engineering, Hefei University of Technology, Hefei 230009, China; 3 College of Materials Science and Engineering, Chongqing University, Chongqing 401133, China

**Keywords:** BMGs, microstructural inhomogeneity, tribological behaviors, HRRF

## Abstract

Microstructural inhomogeneity of bulk metallic glasses (BMGs) plays a significant role in their mechanical properties. However, there is hardly ant research concerning the influence of heterogeneous microstructures on tribological behaviors. Hence, in this research, the tribological behaviors of different microstructural-heterogeneity BMGs sliding in-air were systematically investigated, and the corresponding wear mechanisms were disclosed via analyzing the chemical composition and morphology of the wear track. Higher microstructural-heterogeneity BMGs can possess a better wear resistance both under dry sliding and a 3.5% NaCl solution. The results suggest that microstructural heterogeneity enhancement is a valid strategy to improve the tribological performance of BMGs.

## 1. Introduction

Bulk metallic glasses (BMGs) exhibit superior mechanical and physical properties because of their disordered atomic microstructure [1,2,3]. Consequently, many researchers have been attracted to developing BMGs for their potential applications. However, most scholars focus on the plasticity of BMGs. As a potential structural material, the tribological property is a very significant performance factor in engineering equipment with relative motions in service [4], such as artificial bones [5], golf clubs [6], gear wheels, etc. With very high hardness and elasticity, BMGs are considered superior as wear resistant materials [1]. According to current research, some studies demonstrate that BMGs possess a much longer lifetime in wear applications than crystalline materials [7,8]. For example, A. Inoue et al. reported that micro-sized bearing rollers made of Ni-based BMGs exhibited a lifetime of 2500 h compared with 8 h for SK-steel [9]. However, contradictory conclusions appeared in other experiments [10,11]. For instance, Tam et al. found that Cu-based BMG had a worse frictional coefficient and wear rate than AISI 304 stainless steel under dry and 3.5% NaCl solution, even though Cu-based BMG showed a higher hardness [5]. Additionally, further studies on the atomic-scale and nanoscale scratch wear resistance of a Cu_47_Zr_45_A_l8_ bulk metallic glass by S.V. Ketov et al. identified that the wear rate is found to be significantly reduced by the formation of native and artificially grown surface oxides, indicating that surface oxides hold better wear resistance than Cu4_7_Zr_45_Al_8_ bulk metallic glass [12]. Furthermore, increasing efforts have been made to study the tribological behaviors of BMG under seawater. Since seawater is generally simulated with a 3.5% NaCl solution, the tribological behaviors of BMG should also be studied in 3.5% NaCl [13]. Hence, it is necessary to explore a way to further strengthen BMGs’ wear resistance both under dry sliding and 3.5% NaCl solution.

Nevertheless, contrary to crystalline metallic materials, the brittleness of BMGs could promote crack propagation and aggravate the delamination of the oxide layers during the wear behavior, resulting in a serious weakening of their tribological behaviors [14,15,16]. From this point of view, overcoming brittleness becomes greatly significant. Previous studies demonstrate that fabricating the high microstructural inhomogeneity in BMGs is an emerging strategy for remarkably increasing plasticity [17,18,19].

Inspired by this strategy, we have developed the high rheological rate forming method (HRRF), which could improve plasticity of BMGs by modulating the microstructural heterogeneity of BMGs [17]. Among the various BMGs, Zr-based BMGs have been promising as structural components in many fields due to their high glass-forming abilities, high strength, and high elastic strain [4]. Yet, building on this approach, we changed the microstructural heterogeneity of Zr_54.46_Al_9.9_Ni_4.95_Cu_29.7_Pd_0.99_ BMGs by HRRF to systematically clarify more information between tribological behaviors and the microstructural heterogeneity of BMGs under dry sliding conditions and in 3.5% NaCl solution.

## 2. Materials and Methods

Zr_54.46_Al_9.9_Ni_4.95_Cu_29.7_Pd_0.99_ BMG was prepared through arc melting under a high-purity argon atmosphere. To achieve homogeneity of composition, the master alloys were melted at least four times. Then, a BMG rod with a diameter of 6 mm was produced by copper mold suction casting and cut into a length of 27 mm for HRRF. The wear experiment was performed using an MMW-1A pin-on-disk apparatus in open air under dry conditions and with a 3.5% NaCl solution. BMG samples with a size of φ4 × 13 mm were machined as wear pins to be rubbed against a rotating steel disk of micro-hardness (HV) 728. In the pin-on-disk test, the normal load was 40 N while sliding at a speed of 0.13 m/s, and the sliding duration was 1800 s. The surface microstructure of the samples was recorded using a JSM-6490LV scanning electron microscope (SEM) with energy dispersive X-ray spectrometry (EDS) and a Cypher S atomic force microscope (AFM). All the pin surfaces of samples and disk surfaces were polished using diamond paste. Under each given condition, three samples were measured to ensure the reliability of the data. The glass structure of alloys was confirmed by an X-ray diffractometer (XRD) with Cu Ka radiation. After a wear experiment, each specimen was cleaned ultrasonically. The weight change of each sample before and after the friction tests was determined using an AUY 120 balance with a precision of 0.0001 g. The wear rate was calculated as [20].

(1) $$W=\frac{V}{F}\times S$$

where  V is the wear volume loss of the sample, F is the applied normal load, and S is the total sliding distance.

The nano-indentations were carried out over a square area of 48 × 48 μm^2^ for the as-cast, and samples were treated with a Berkovich diamond tip. The constant depth was 300 nm. Each nano-indentations were penetrated at the same depth (300 nm) at a constant loading rate of 0.05 s^−1^, and the spacing between adjacent indentations was 6 μm.

The HRRF was introduced to manipulate the microstructural heterogeneity of BMGs. As reported in our previous study [19], this fabrication process consists of three steps, heating up to the supercooled liquid region through fast Joule heating, squeezing the supercooled liquid into a copper mold cavity under the preset load, and then rapidly cooling it down to room temperature in the copper mold.

## 3. Results and Discussions

### 3.1. Microstructure

**Figure 1:**
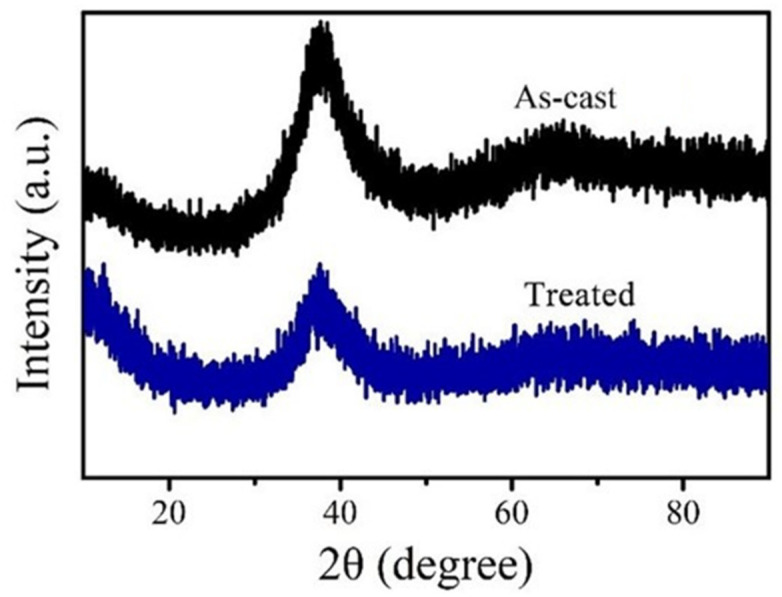
XRD patterns of Zr_54.46_Al_9.9_Ni_4.95_Cu_29.7_Pd_0.99_ BMGs before and after HRRF treatment [17].

Figure 1 illustrates the X-ray diffraction pattern of as-cast and treated Zr_54.46_Al_9.9_Ni_4.95_Cu_29.7_Pd_0.99_ BMGs. It shows a broad halo with the absence of detectable crystalline peaks, indicating that the treated sample remained a glassy structure.

**Figure 2:**
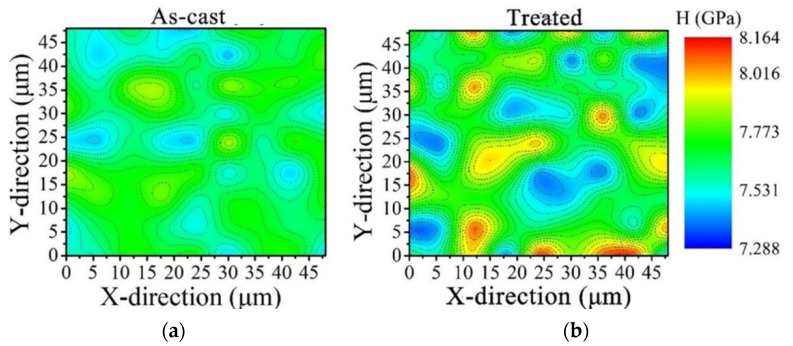
(**a**,**b**) are the distribution of the nano-indentation hardness of the as-cast [17] and treated sample, respectively.

To gain a better understanding of heterogeneous microstructure for as-cast and treated Zr_54.46_Al_9.9_Ni_4.95_Cu_29.7_Pd_0.99_ BMGs, the distributions of the nano-hardness were obtained, as shown in Figure 2. It is obvious that the as-cast material is rather homogeneous, with hardness values ranging between 7.45 and 7.91 GPa. Conversely, the treated sample is much more heterogeneous and displays a wider range of hardness values (7.288–8.164 GPa). To a certain extent, these distributions demonstrated that the microstructure of treated BMGs can be considered more inhomogeneous than the as-cast samples. Confirmed by early studies, this phenomenon can be interpreted by Lennard-Jones-like potential function. During HRRF-treating, the drastic amount of mechanical work by the preset load was intruded into BMGs driving short-range atomic rearrangement [17,20], leading to hard regions with a higher atomic packing density, and soft regions with a lower atomic packing density.

### 3.2. Roughness of Pre-Tested Surfaces

**Figure 3:**
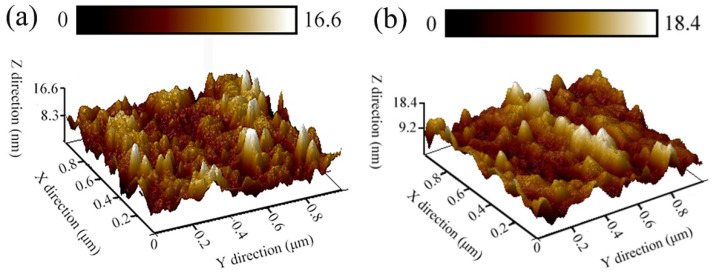
(**a**,**b**) are are high-magnification detailed micrographs of pre-tested surfaces for as-cast and treated samples to be tested under AFM.

It is well known that roughness of pre-tested surfaces would interfere with the friction properties of BMGs [21]. Therefore, before friction tests, AFM are used to evaluate pre-tested surfaces. The micro-morphology, pre-tested surfaces of the as-cast and treated BMGs are analyzed by AFM as illustrated in Figure 3a,b. It is intuitively found that all pre-tested surfaces exhibit the interlaced topography of “peak” and “valley” on the pre-tested surfaces. Significantly, the relative width of the pre-tested surface roughness for the as-cast glass is 16.6 nm, much the same as that (18.4 nm) in the treated sample. Moreover, by further calculation, the average surface roughness of pre-tested surfaces (Ra) of the cast sample is 4.61 nm, and the Ra of the treated sample is 4.9 nm. Such tiny changes suggest that the pre-tested surfaces exhibit ideal flatness, minimizing the effect of the surface roughness of pre-tested surfaces on the accuracy of friction experiments.

### 3.3. Wear Performance

**Figure 4:**
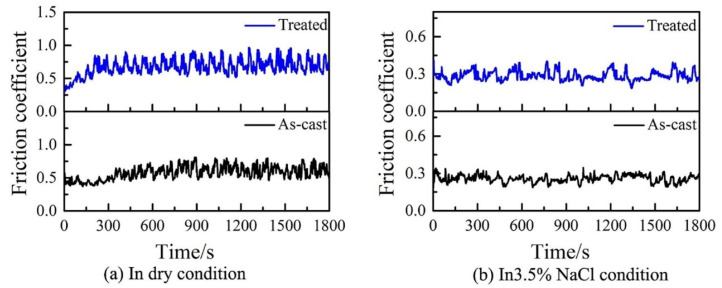
The friction coefficient of as-cast and treated Zr_54.46_Al_9.9_Ni_4.95_Cu_29.7_Pd_0.99_ BMGs as a function of sliding time under (**a**) dry and (**b**) 3.5% NaCl solution.

As shown in Figure 2, HRRF could enhance the microstructural heterogeneity of Zr_54.46_Al_9.9_Ni_4.95_Cu_29.7_Pd_0.99_ BMGs. Thus, the as-cast and treated BMGs were tested for wear resistance. Figure 4 presents the friction coefficient curves of as-cast and treated BMGs under dry conditions and 3.5% NaCl solution. As described in Figure 4a, it was found that both curves have a steady-state stage after an initial rapid increasing period under the dry-friction condition; the coefficients of friction in the steady-state stage are ~0.578 and ~0.676 for as-cast and treated samples respectively. The treated BMG displays a higher friction coefficient (COF). This phenomenon may be interpreted as that during the friction experiment, the harder regions in treated BMGs can remain intact for a long time, which increases the relative movement resistance between the material and the friction pair [22]. However, in Figure 4b, under the 3.5% NaCl solution condition, the friction coefficients of all samples exhibit around 0.29, and much lower, values than those in the air condition, owing to the lubrication effect of the NaCl solution.

**Figure 5:**
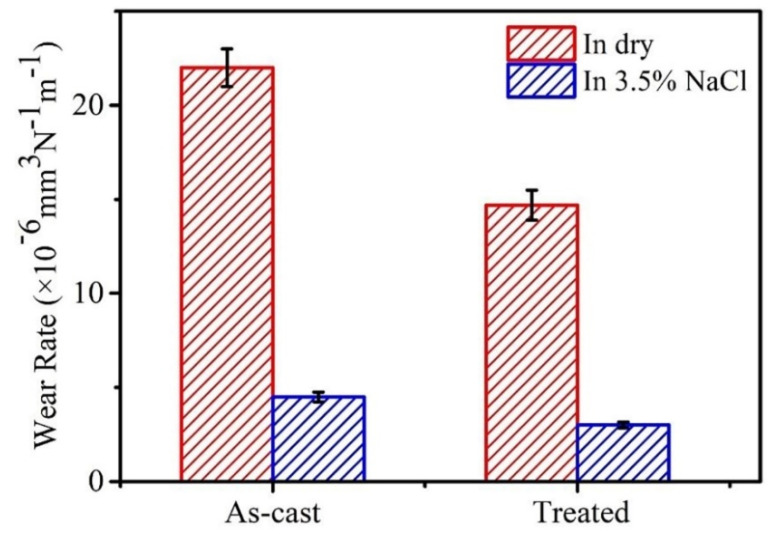
The wear rate of as-cast and treated Zr_54.46_Al_9.9_Ni_4.95_Cu_29.7_Pd_0.99_ BMGs in different environments.

The wear rate is always regarded as an important parameter to evaluate the wear resistance of the materials [6,23]. Thus, the wear rate of as-cast and treated BMGs under dry and 3.5% NaCl solution are illustrated in Figure 5. The wear rates of as-cast BMG and treated BMG in dry are 22.1 × 10^−6^ mm^3^·N^−1^m^−1^ and 14.7 × 10^−6^ mm^3^·N^−1^m^−1^, respectively. In addition, the wear rates of BMGs sliding in 3.5% NaCl solution decreased in the following order: 4.5 × 10^−6^ mm^3^·N^−1^m^−1^ for the as-cast BMG, and 3 × 10^−6^ mm^3^·N^−1^m^−1^ for the treated BMG. Evidently, the values of wear rates of as-cast samples show remarkably higher values than those of the treated samples under different conditions.

To be specific, under the dry conditions, due to the direct contact friction between the friction pair of metals, the friction-induced heat easily appears on the tested surfaces of samples [24]. With the heat accumulated constantly, the worn surface not only becomes soft, but also reacts with air to form oxide layers. The hardness of the oxide layers is higher than that of the BMGs, thereby improving the wear resistance of the material. However, due to the high hardness and limited ductility of the oxide layer, it is easy to induce the separation of the oxide layer from the BMGs matrix under the action of sliding shear force, which generates oxide layers which can wear out more easily than BMGs [25,26]. BMGs treated by HRRF would show a supernal microstructural inhomogeneity, which indicates that the hard regions become stronger and the soft regions become more fragile compared with the as-cast BMGs. Hence, for treated BMGs, because of the higher-density arrangement of atoms, it is difficult to oxidize the hard regions. As a result, the number of oxide layers generated on the worn surface of the treated sample may be less than those of the as-cast sample. Eventually, the treated BMGs exhibit higher wear resistance. Similarly, under 3.5% NaCl solution, although there are only a few oxide layers on the worn surfaces of BMGs for a good cooling effect of the solution, due to harder regions, the treated BMGs still show a better wear resistance ability. All results evidence that enhancing the microstructural inhomogeneity of BMGs can improve the wear resistance effectively in these two external environments.

### 3.4. Worn Surfaces Analysis

**Figure 6:**
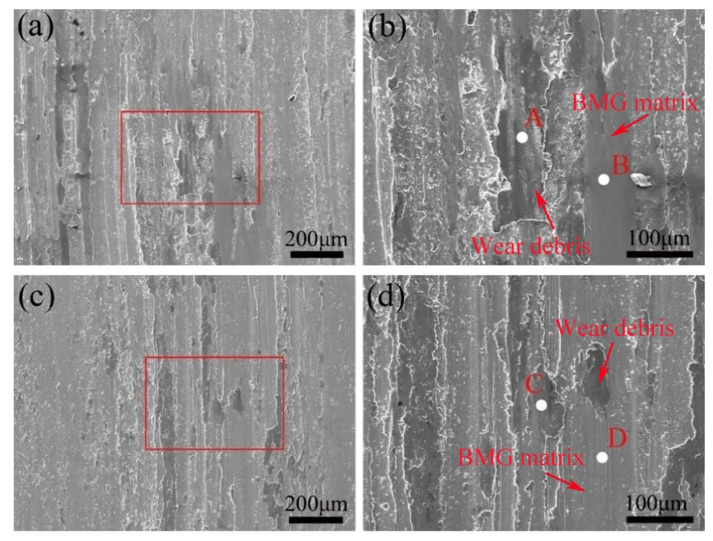
(**a**,**c**) SEM images of worn scars of as-cast and treated Zr_54.46_Al_9.9_Ni_4.95_Cu_29.7_Pd_0.99_ BMGs tested in dry-sliding, respectively. (**b**,**d**) High-magnification detailed micrographs corresponding to the part marked by the red-lined rectangular in (**a**,**c**), respectively.

To intuitively understand the differences in wear performance between the as-cast and treated BMGs, the morphologies of the wear scars were further examined by the SEM. Figure 6a,c display the worn surface micrographs of as-cast and treated BMGs under dry conditions; the deep and wide grooves with flake-like wear debris parallel to the sliding direction can be clearly observed on the worn surfaces of all BMGs, which suggests that the wear mechanism is mainly controlled by the integration of abrasive wear and adhesive wear [27]. Apparently, at the beginning of the dry-sliding, many wear debris particles appear between the friction pair and the sample. This debris may act as abrasive particles to plow the renewed surface, trap between the contact surfaces, and squeeze into the subsurface, resulting in the formation of ploughed grooves [28]. As the friction test goes on, these wear debris particles can be gradually converged and softened (or liquefied) once the temperature of frictional heating on the wear surface is elevated close to the temperature of glass transition. Eventually, they turn into large, flake-like wear debris which attaches to the wear surface of as-cast and treated samples. Furthermore, upon closer inspection, there are serious delamination, peeling, and micro-cracks on the wear surface of as-cast samples (as illustrated in Figure 6b), indicating that the highly localized stress concentration is induced on this surface on account of the brittleness of as-cast BMGs [29]. However, as shown in Figure 6d, there is no delamination or cracks appearing on the worn surfaces of treated BMGs, due to the higher inhomogeneous structure which would possess the higher ductility. When subjected to compressive stress or shear stress during the process of friction, the treated samples are more likely to undergo plastic deformation, effectively avoiding the appearance of delamination, peeling, and cracks.

**Figure 7:**
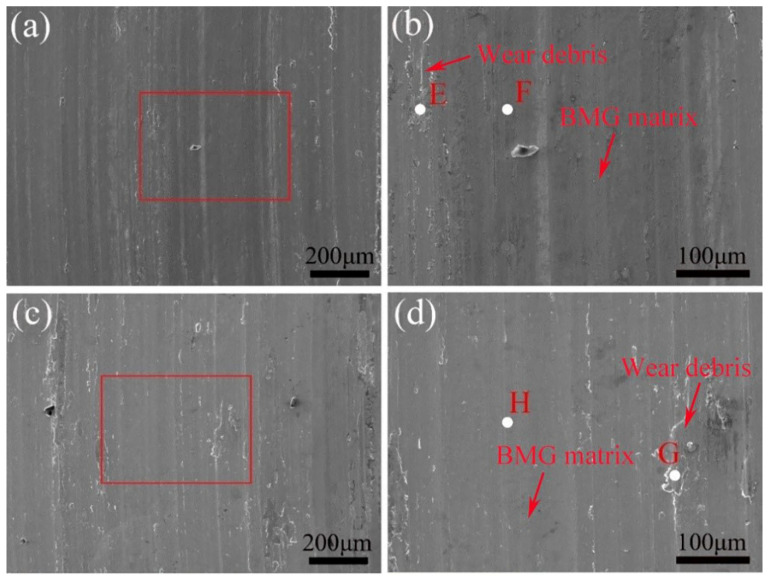
(**a**,**c**) SEM images of worn scars of as-cast and treated BMGs tested in 3.5% NaCl solution, respectively. (**b**,**d**) High-magnification detailed micrographs corresponding to the part marked by the red-lined rectangular in (**a**,**c**), respectively.

Figure 7a,c display the worn surfaces of as-cast and treated BMG in 3.5% NaCl solution, respectively, at low magnification. Owing to the lubricating effect of NaCl solution [30], these worn surfaces are covered with several narrow and shallow grooves and a few flake-like wear debris, which appear relatively smoother than those under dry sliding conditions, indicating slight abrasive and adhesive wear. Meanwhile, in order to observe the differences in the worn surface morphology of as-cast and treated BMGs more clearly, Figure 7b,d show detailed micrographs corresponding to the red-lined rectangular in Figure 7a,c respectively. Apparently, the wear debris on the worn surfaces of the treated sample are very similar to those of the as-cast sample.

**Figure 8:**
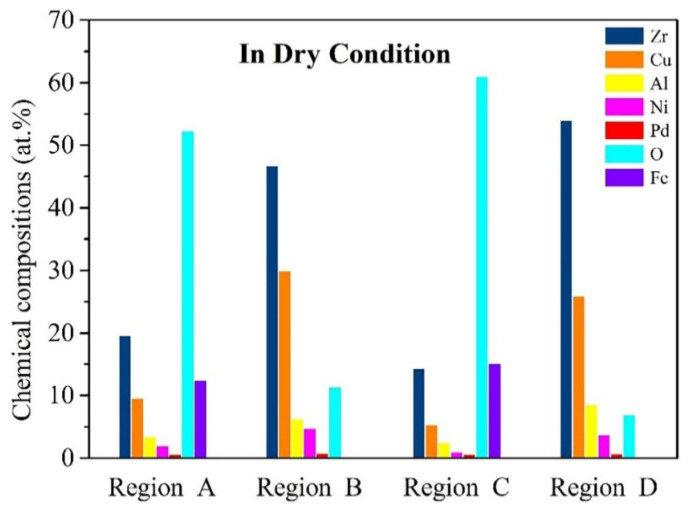
Chemical compositions of typical regions on the worn scar of as-cast and treated BMGs in dry-sliding.

In addition to the worn surface morphology, the residual elements on the worn surface, which come either from the surrounding environment or the counterpart, are also an important basis for understanding the friction process [31]. Therefore, a SEM-EDS experiment was carried out. Figure 8 shows the SEM-EDS point spectrum of wear debris regions (marked by A in Figure 6b and C in Figure 6d and BMG matrix regions (marked by B in Figure 6b and D in Figure 6d). The Figure 8 shows that the oxygen appears on the worn surfaces of all samples, which confirms our previous supposition that surface oxidation takes place during the friction process [32]. Further observing BMG matrix regions, the oxygen content of the as-cast sample is 11.4%, while the treated sample is just 7%, suggesting that harder regions make it more difficult for the treated samples to participate in the oxidation reaction. Whereas as shown in wear debris areas, it is interesting to note that both as-cast and treated samples exhibit a larger amount of oxygen (over 50 at%) and the iron element, indicating that the wear debris would not only react violently with oxygen in the air, but also cause material transfer between BMGs and friction pairs. Furthermore, by comparing the variation in the chemical compositions of region A and region C, the oxygen content of wear debris increased from 52.3% in the as-cast BMGs to 61.3% of the treated sample, and the Fe content increased from 12.5% in the as-cast state up to 15.2%, contrary to the tendency of BMG matrix regions. This may be explained by the wear debris materials from the soft regions of worn surfaces; greater softness of the soft regions for treated BMGs results in their wear debris being more inclined to actively participate in the reaction.

**Figure 9:**
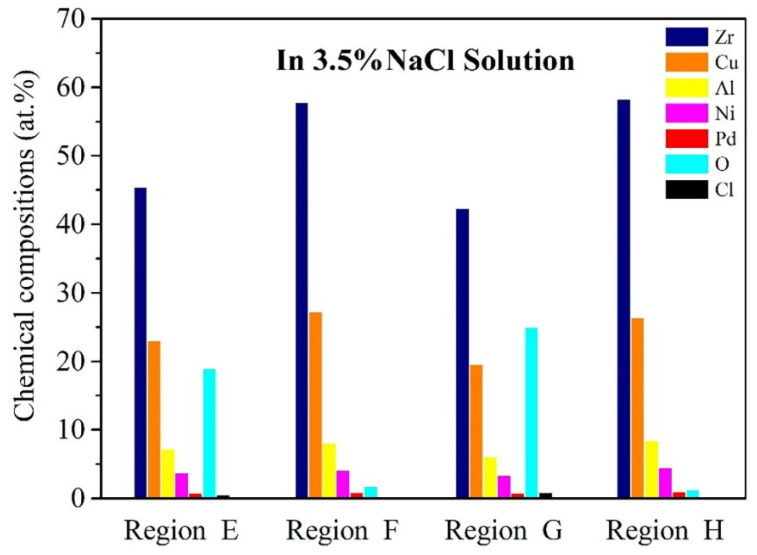
Chemical compositions of typical regions on the worn scar of as-cast and treated BMG in 3.5% NaCl solution respectively.

As illustrated in Figure 9, the EDS analysis was performed under 3.5% NaCl solution on wear debris regions (marked by E in Figure 7b and G in Figure 7d) and BMG matrix regions (marked by F in Figure 7b and H in Figure 7d). It is obvious that the extremely low content of oxygen on the as-cast and treated BMG matrix regions is less than 2%, showing that the NaCl solution can reduce friction heat effectively, which minimizes the probability of an oxidation reaction on the friction surface [31]. Moreover, the chlorine element was not detected in this area, indicating that the corrosive effect of chloride ions could not have a significant impact on this area. Conversely, when observing the point scanning results of the wear debris regions, it is worthwhile to note the higher oxygen content and the appearance of chlorine elements. This implies that the wear debris is more easily oxidized and corroded as well, under the 3.5% NaCl solution. Furthermore, the contents of O and Cl on the wear debris of as-cast samples are both lower than the treated samples.

## 4. Conclusions

In summary, our observations revealed that wear resistance behaviors under the dry and the 3.5% NaCl solution conditions can be improved by imparting high structural heterogeneity to Zr_54.46_Al_9.9_Ni_4.95_Cu_29.7_Pd_0.99_ BMG using the HRRF method. Under the dry condition, it is obvious that BMGs with different levels of structural heterogeneity showed significantly different wear resistance behaviors. The treated BMG exhibited a wear rate of ~14.7 × 10^−6^ mm^3^·N^−1^m^−1^ which is superior to the ~22.1 × 10^−6^ mm^3^·N^−1^m^−1^ wear rate of the as-cast BMG. Moreover, the predominant wear mechanism of all BMGs under dry-sliding conditions is characterized as abrasive and oxidation wear. Additionally, under the 3.5% NaCl solution condition, the main wear mechanisms are abrasive wear, slight corrosion wear, and adhesive wear. Due to the lubrication and cooling from the solution, although the wear rate of BMGs exhibits much lower levels than those of the dry-friction condition, the wear rate of treated BMGs (~3 × 10^−6^ mm^3^·N^−1^m^−1^) was also below the as-cast samples (~4.5 × 10^−6^ mm^3^·N^−1^m^−1^). Hence, the combination of improved wear resistance of BMGs both in dry and 3.5% NaCl solution conditions can be achieved. On one hand, the number of oxide layers generated on the worn surface of the treated sample were less than those of the as-cast sample. On the other hand, the higher inhomogeneous structure possesses higher ductility. It can effectively avoid the appearance of delamination, peeling, and cracks on the worn surface. This research provides an unprecedented perspective on BMGs, with easy implementation and celerity to improve wear resistance. We believe our approach presents a guide for the future development of BMGs.
